# The Interface between Fungal Biofilms and Innate Immunity

**DOI:** 10.3389/fimmu.2017.01968

**Published:** 2018-01-10

**Authors:** John F. Kernien, Brendan D. Snarr, Donald C. Sheppard, Jeniel E. Nett

**Affiliations:** ^1^Department of Medicine, University of Wisconsin, Madison, WI, United States; ^2^Department of Microbiology and Immunology, McGill University, Montreal, QC, Canada; ^3^Department of Medicine, McGill University, Montreal, QC, Canada; ^4^Department of Medical Microbiology and Immunology, University of Wisconsin, Madison, WI, United States

**Keywords:** biofilm, matrix, fungi, neutrophil extracellular trap, innate immunity, neutrophil, *Aspergillus*, *Candida*

## Abstract

Fungal biofilms are communities of adherent cells surrounded by an extracellular matrix. These biofilms are commonly found during infection caused by a variety of fungal pathogens. Clinically, biofilm infections can be extremely difficult to eradicate due to their resistance to antifungals and host defenses. Biofilm formation can protect fungal pathogens from many aspects of the innate immune system, including killing by neutrophils and monocytes. Altered immune recognition during this phase of growth is also evident by changes in the cytokine profiles of monocytes and macrophages exposed to biofilm. In this manuscript, we review the host response to fungal biofilms, focusing on how these structures are recognized by the innate immune system. Biofilms formed by *Candida, Aspergillus*, and *Cryptococcus* have received the most attention and are highlighted. We describe common themes involved in the resilience of fungal biofilms to host immunity and give examples of biofilm defenses that are pathogen-specific.

## Introduction

**Figure 1:**
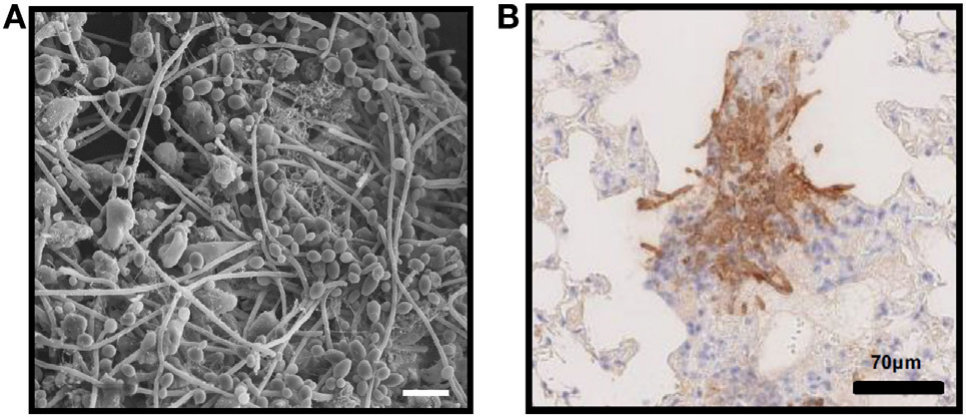
*In vivo* fungal biofilms. **(A)** *Candida albicans* biofilm growing on the luminal surface of a rat venous catheter for 24 h. Scanning electron microscopy reveals adherent organisms growing within in an extracellular matrix. **(B)** Immunohistochemistry of pulmonary tissue from an immunocompromised mouse infected with *Aspergillus fumigatus* and stained with an anti-galactosaminogalactan antibody. Brown indicates accumulation of galactosaminogalactan-containing biofilm matrix surrounding hyphae growing within pulmonary tissues.

Fungi frequently flourish as biofilms, which are aggregated communities encased in a protective extracellular matrix (1, 2). Many clinically relevant fungi have been shown to form biofilms, such as *Candida* spp., *Aspergillus* spp., *Cryptococcus neoformans, Fusarium* spp., *Blastoschizomyces capitatus, Malassezia pachydermatis, Pneumocystis* spp., *Trichosporon asahii, Rhizopus* spp., and *Rhizomucor* spp (3–13). In the clinical setting, fungal biofilms can propagate on artificial medical devices, such as catheters, as well as on epithelial and endothelial surfaces (3, 14–19) (Figure 1A). In addition, during invasive infection, fungal pathogens can proliferate as non-surface associated microcolonies embedded in extracellular matrix (18) (Figure 1B). Biofilms resist antifungal therapies and host defenses, making them notoriously difficult to eradicate (4, 20–36). One defining trait of biofilm formation is the production of a microbial-produced extracellular matrix, or the “glue” necessary for adhesion, which also serves as a shield that creates protected reservoirs of infection (4, 5, 20, 33, 37). As investigations reveal the complex composition of the extracellular matrix for several fungal pathogens, it has become increasing clear that this material is distinct from the cell wall (4, 5, 18, 38–41). Therefore, biofilm growth provides a means to present unique moieties and conceal cell wall ligands. Studies are just beginning to shed light on how biofilm formation and matrix production influence host recognition (5, 15, 27, 29–32, 42–50). In this review, we examine the innate immune response to fungal biofilms, highlighting how production of the extracellular matrix alters immunity. While a variety of fungal pathogens have been shown to produce biofilms, investigations examining host interactions have primarily utilized model pathogens *Candida albicans, Aspergillus fumigatus*, and *C. neoformans*, which will be the focus of our discussion.

## *Candida* Biofilms

### Biofilm Formation

**Figure 2:**
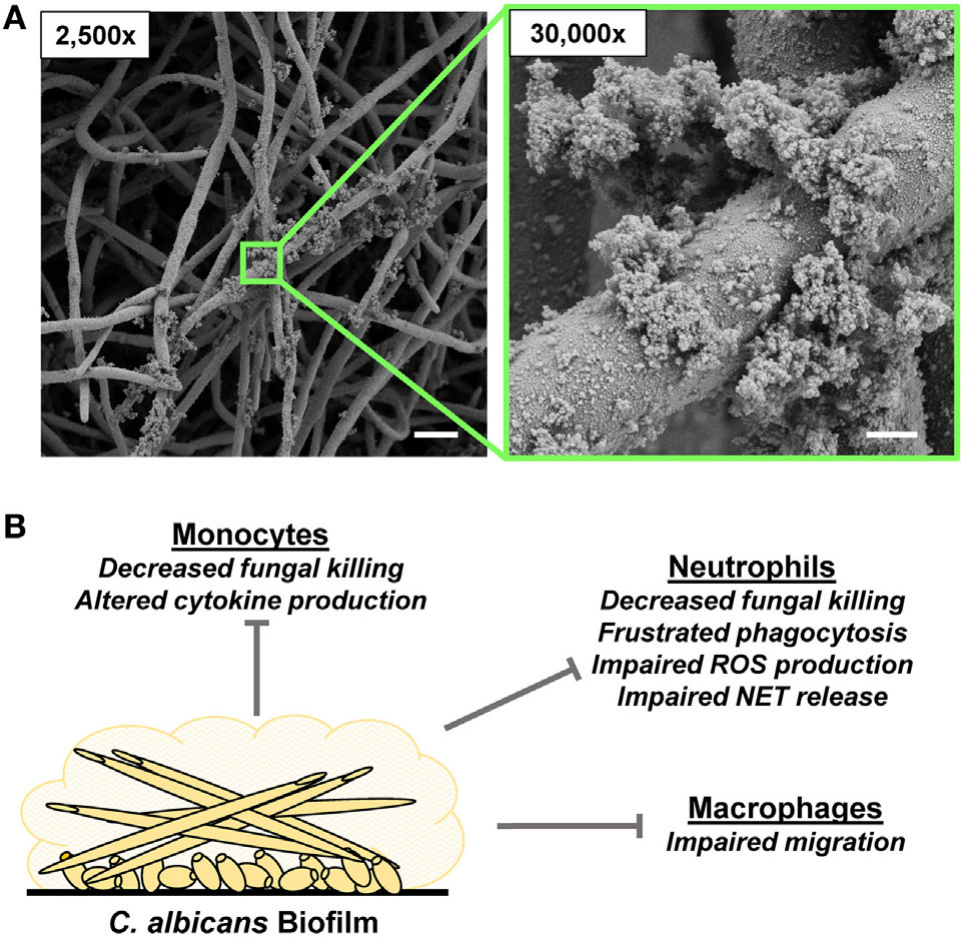
*Candida albicans* biofilm formation and innate immune response. **(A)** Scanning electron microscopy images reveal *C. albicans* biofilms grown on coverslips. Biofilms were grown for 48 h. Measurement bars represent 10 and 1 µm for 2,500× and 30,000×, respectively. **(B)** Summary of innate immune responses impaired by *C. albicans* biofilms.

*Candida* spp., commensal fungi of the gastrointestinal tract, can cause severe, disseminated disease with high mortality, particularly in patients with implanted medical devices or compromised immune systems (17, 51–57). The vast majority of these infections involve biofilm formation on either an artificial or a biotic surface (13, 58, 59) (Figure 2). Clinical *Candida* biofilms grow on diverse medical devices, including central venous catheters, urinary catheters, prosthetic valves, left ventricular assist devices, and oral devices, such as dentures (3, 60). Both vaginal and oral mucosal surfaces promote biofilm formation as well (16, 61). The majority of the *in vitro* and *in vivo* biofilm studies have utilized *C. albicans*, the most widespread species. However, non-*albicans* species, including *Candida tropicalis, Candida parapsilosis*, and *Candida glabrata*, similarly produce clinically relevant biofilms (3, 62–68). In addition, this virulence trait has been described for the emerging pathogen *Candida auris* (69). *Candida* biofilm formation involves the adherence of yeast to a substrate, the proliferation of cells to form a fungal community, and the production of an extracellular matrix (37, 70–72). *C. albicans* biofilm development often involves the production of hyphae, although the degree of filamentation varies among strains and niches. Non-*albicans* strains lacking the ability to filament produce biofilms composed of layers of yeast embedded in an extracellular matrix (73).

### Matrix Composition

Upon encounter with biofilm, immune cells are first confronted with the extracellular matrix covering the fungal cells. A combination of both *in vitro* and *in vivo* models has been integral for the dissection of *Candida* biofilm matrix assembly and composition (20, 22, 39, 40, 58, 67, 70, 74–79). For *C. albicans, in vitro* studies have revealed that the mature biofilm matrix consists of a variety of macromolecules, including protein (55%), carbohydrate (25%), lipid (15%), and DNA (5%) (23, 38, 40, 80). Interestingly, many of the matrix components vastly differ from the cell wall components that would be initially recognized by immune cells in the absence of biofilm (40). For example, the main polysaccharide of *C. albicans* biofilm matrix is α-1,2-branched α-1,6 mannan, which is found in high molecular weight structures of approximately 12,000 residues. In contrast, mannans of the outer cell wall layer are 5- to 10-fold smaller. Furthermore, the matrix mannans associate with β-1,6 glucans, forming a mannan–glucan complex that assembles extracellularly, and this structure has not been identified in the cell wall of *C. albicans* (40, 75). In addition, matrix β-1,6 glucan exists in a linear conformation, while the β-1,6 glucan of the cell wall is highly branched (81). Proteomic analysis of *in vitro* biofilms shows some similarities between matrix-associated proteins and those released into the media during planktonic growth (79). However, the extracellular matrix lacks many of the proteins associated with the cell wall (40, 82). *In vivo*, host proteins contribute to the construction of biofilm, with an astonishing majority (>95%) of the matrix-associated proteins of host origin (76). This finding demonstrates variation in the content of fungal biofilm matrix *in vivo* and highlights the importance of including animal models for investigation of *Candida* biofilms.

### Neutrophil–*Candida* Biofilm Interactions

Neutrophils are primary responders to *C. albicans* infection, with neutropenic patients prone to severe, lethal candidiasis (83–86). Neutrophils respond to chemokines and other signals during recruitment to the site of infection. Upon pathogen encounter, neutrophils elicit various responses important for control of infection, including phagocytosis, degranulation, reactive oxygen species (ROS) production, and neutrophil extracellular trap (NET) release (87). In the context of *C. albicans* biofilms, neutrophils are the primary leukocyte recruited to the site of infection (15, 76, 88–90). In fact, neutrophil recruitment has been observed in animal models mimicking diverse clinical biofilms, including catheter-related infections (vascular and urinary), denture stomatitis, oral candidiasis, and vaginal candidiasis (15, 76, 91). Despite their presence at the site of infection, neutrophils fail to eradicate *Candida* biofilms. Pioneering studies with human neutrophils revealed that *C. albicans* biofilms resist neutrophil attack, in comparison to their planktonic counterparts (29, 30). Intact biofilm structure is required for this resistance, as resuspension of the biofilm cells reverses the phenotype (29). Furthermore, biofilm impairment of neutrophils is robust, persisting despite neutrophil priming by IFN-γ or G-CSF (92).

The ability of *Candida* biofilms to withstand immune attack appears to vary by strain and species. For *C. albicans*, biofilms are approximately twofold to fivefold more resistant to killing when compared to planktonic cells (29–31, 92). Investigation of *C. parapsilosis* found a similar trend, but did not identify a significant difference in susceptibility to neutrophils between biofilms and planktonic organisms (43, 93). The lack of statistical significance was attributed to the heterogeneity of biofilm formation, resulting in high assay variability. A recent investigation found *C. glabrata* biofilms also resist neutrophil killing, exhibiting a threefold higher resistance for biofilm over planktonic organisms (45).

For killing and containment of *C. albicans*, neutrophils release NETs, which are structures of DNA, histones, and antimicrobial proteins (94, 95). These structures are particularly well-suited to combat large organisms, such as hyphae, which are unable to be ingested by phagocytosis (95). As *C. albicans* biofilms consist of aggregated cells and hyphal elements, NETosis would seemingly be an efficient method of attack. However, a recent study has revealed that neutrophils fail to release NETs in the presence of *C. albicans* biofilms (31). This phenomenon is conserved across a variety of *C. albicans* strains exhibiting differing degrees of filamentation and biofilm architecture (44). This inhibitory pathway appears to be closely linked to the production of an extracellular matrix, as physical or genetic disruption of this process restores NET release (31). Remarkably, when neutrophils are induced to generate NETs prior to biofilms exposure, biofilm inhibition is observed (31). This suggests that inhibition of NETosis is an adaptation by *C. albicans* biofilms to prevent killing by neutrophils. Other species appear to employ this mechanism as well. For example, *C. glabrata* biofilms also impair NET release, although the inhibition is not as pronounced (45).

Recent studies have begun to shed light on the planktonic *C. albicans* cell surface components that induce NET release. The process appears to be multifactorial, as β-glucan, mannan, and secreted aspartic proteases all variably trigger NETosis (93, 96, 97). NET inhibition by biofilm likely involves concealment of cell surface ligands by the extracellular matrix, as disruption of this process permits NET release (31). In particular, disruption of the matrix mannan–glucan complex in a *pmr1*Δ/Δ mutant strain reverses the NET inhibition phenotype, suggesting a role for this unique polysaccharide complex (31, 75). In addition, studies by Zawrotniak et al. show that NET induction by cell wall mannan is concentration-dependent, with higher concentrations failing to trigger NETosis *in vitro* (97). Further investigation would be of interest to explore a similar pattern for polysaccharides of the biofilm matrix in NET inhibition.

Upon encounter with *C. albicans* biofilms, the generation of ROS by neutrophils is dampened compared to the response observed for planktonic organisms (30, 31, 44). Multiple pathways govern NET release and a subset of these depend on ROS production (96, 98–103). In response to planktonic *C. albicans*, both ROS-dependent and ROS-independent pathways trigger NETosis, which may not be surprising given the numerous cell surface ligands expected to be involved (96, 97, 103). While further investigation is needed to dissect pathways impairing neutrophil function by biofilms, inhibition of ROS production is likely involved. A study of *C. glabrata*–neutrophil interactions demonstrates a similar neutrophil response, with reduced ROS production upon encounter with biofilm (45). Taken together, these studies show that *C. albicans* biofilms inhibit the release of NETs and resist killing by neutrophils. The pathway appears to involve the production of an extracellular matrix and dampening of neutrophil ROS production. Based on studies with *C. glabrata*, it may be conserved, in part, among *Candida* spp.

### Monocytes and Macrophages Interactions with *Candida* Biofilms

Chandra et al. first demonstrated that *Candida* biofilms resist attack by monocytes and can alter their cytokine profile (42). Peripheral blood mononuclear cells (PBMCs) fail to phagocytose biofilm-associated *C. albicans*, in contrast to planktonic organisms (42). However, these cells remain viable, migrating within the biofilm, even providing a stimulus for biofilm proliferation through an unknown mechanism (29, 42). Compared to planktonic organisms, *C. albicans* biofilms are twofold to threefold more resistant to killing by monocytes (29).

Encounter with *Candida* biofilms influences cytokine release by mononuclear cells. One of the more intriguing alterations is the downregulation of TNF-α, a cytokine which facilitates phagocyte activation. Compared to planktonic organisms, exposure to *C. albicans* biofilms significantly diminishes the production of TNF-α by monocytic cell line THP-1 (29). Not only is this predicted to impact phagocyte function in the host but the alteration in production of TNF-α may also have a direct impact on the biofilm. Application of exogenous TNF-α has been shown to prevent *C. albicans* biofilm formation, through a TNF receptor-independent pathway (104). Furthermore, this activity is blocked by preincubation of TNF-α with *N*,*N*′-diacetylchitobiose, a major carbohydrate component of *C. albicans* cell wall (104). Therefore, inhibition of TNF-α by biofilms may represent an evolutionary adaption and mechanism of immune evasion. However, much remains a mystery about how the cytokine response influences the host response to biofilm infection. For example, when compared to planktonic cells, PBMCs exposed to *C. albicans* biofilms produce elevated levels of IL-1β, IL-10, and MCP-1 and reduced levels of IL-6 and MIP1β (42). How these combinations of both pro- and anti-inflammatory cytokines are triggered and their influence on host response to biofilm infection is unknown.

Recent work by Alonso et al. revealed that formation of *C. albicans* biofilm impairs the migratory capacity of macrophages (105). Upon exposure to biofilm, the migration of murine macrophages (J774.1 cell line) is reduced approximately twofold when compared to encounter with planktonic organisms. A *pmr1*Δ/Δ mutant similarly impaired macrophage migration during biofilm growth. As this biofilm is deficient in matrix mannan production, the macrophage inhibition is likely related to another factor, such as physical structure (75, 105). Therefore, *Candida* biofilms may elicit distinct inhibitory pathways for neutrophils and macrophages (31, 105).

## *Aspergillus* Biofilms

### Biofilm Formation

**Figure 3:**
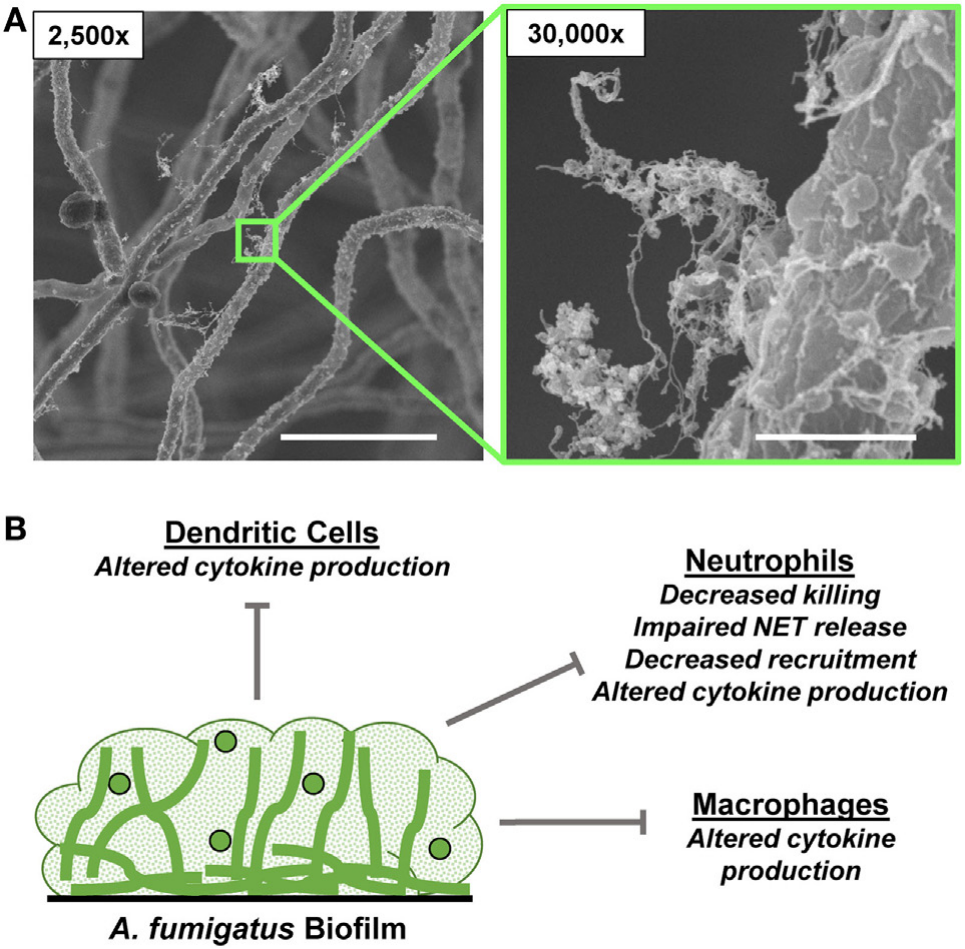
*Aspergillus fumigatus* biofilm formation and innate immune response. **(A)** Scanning electron microscopy images reveal *A. fumigatus* biofilms grown on coverslips. Biofilms were grown for 24 h. Measurement bars represent 10 and 1 µm for 2,500× and 30,000×, respectively. **(B)** Summary of innate immune responses impaired by *A. fumigatus* biofilms.

*Aspergillus* spp. grow ubiquitously in the environment and individuals are constantly exposed to their spores, which are released into the air (106). Immunocompetent individuals clear these spores after inhalation, but those with impaired immunity are at risk for development of severe disease. *Aspergillus* spp. can cause a variety of clinical diseases, including invasive, chronic, and allergic forms (106). The chronic form of disease typically involves formation of an aspergilloma, or fungal ball, in the sinus or lung cavity. These dense structures consist of agglutinated hyphae with occasional conidial heads growing as a biofilm encased in an adhesive extracellular matrix (18) (Figure 3). As the community matures, the inner cells loose viability, likely due to starvation. *A. fumigatus* also produces extracellular matrix material during invasive pulmonary aspergillosis (18). However, during this mode of growth, the hyphae remain separated without an inner core of decaying fungal mass. *In vitro, A. fumigatus* forms biofilms on agar medium in aerial, static conditions that mimic the host niches for aspergilloma formation (4).

### Matrix Production

*Aspergillus fumigatus* produces a unique extracellular matrix during biofilm growth *in vitro* and *in vivo* (4, 18, 107). By biochemical analysis, this material consists of 40% protein, 43% carbohydrates, 14% lipids, and 3% aromatic-containing compounds, as well as DNA (108–110). The polysaccharides of the extracellular matrix exhibit cohesion properties and provide immune protection. The main matrix polysaccharides include galactomannan and galactosaminogalactan (GAG), of which, GAG has received the most attention (18). *A. fumigatus* strains deficient in GAG production lack the capacity to form biofilms or produce extracellular matrix (47). GAG is an α-1,4-linked linear heteroglycan composed of variable combinations of galactose and *N*-acetyl-galactosamine (GalNAc) (111, 112). GalNAc residues within the GAG polymer are partially deacetyated by the secreted enzyme Agd3, rendering mature GAG polycationic (113). Deacetylation is required for GAG to mediate adhesion between hyphae and other anionic surfaces such as host cells, plastic, and glass (113). GAG production has also been reported for other *Aspergillus* spp., including *Aspergillus parasiticus, Aspergillus niger*, and *Aspergillus nidulans*, although the relative proportion of galactose and GalNAc varies between strains and likely influences matrix function as detailed below (114–117). While galactomannan and GAG are universally present in *A. fumigatus* matrix, key differences exist between the clinical niche biofilms. For example, aspergilloma biofilms produce a thicker extracellular matrix, which contains α-glucan, a polysaccharide absent in biofilms formed during invasive aspergillosis (18). Aspergilloma biofilms also produce melanin, an immune modulator (4, 18, 33, 118). However, its specific role during biofilm formation is unknown.

### Innate Immunity to *Aspergillus* Biofilms

Neutrophils are key players in the innate immune defense against *Aspergillus*. Neutropenia, often in the face of chemotherapy or hematologic malignancy, places patients at high risk factor for invasive pulmonary aspergillosis, which can progress to disseminated lethal disease (119, 120). Neutrophils are recruited to *Aspergillus* spores *in vivo* and are critical for their engulfment through phagocytosis (121–123). Neutrophils also release NETs in response to hyphal elements (46, 124–126). While NETs lack significant activity against conidia, they exhibit modest inhibitory activity against the larger hyphal forms of *A. fumigatus* (46, 126). Production of an extracellular matrix shields *A. fumigatus* from neutrophil attack and much of this protection is attributed to GAG (32). *A. fumigatus* mutants deficient in GAG synthesis or deacetylation exhibit attenuated virulence (47, 113), and treatment with recombinant glycoside hydrolases that degrade GAG reduces fungal growth in a murine model of aspergillosis (127).

Studies of differences in GAG composition between *A. fumigatus* and *A. nidulans* have suggested that protection against neutrophil attack is mediated by hyphal-associated GalNAc-rich GAG (32). Unlike *A. fumigatus, A. nidulans* produces GalNAc-poor GAG, which contains over fivefold higher levels of galactose and produces poorly adherent biofilms containing minimal extracellular matrix. *A. nidulans* is also less virulent in a murine model of pulmonary aspergillosis and more than twofold more susceptible to killing by human neutrophils. Heterologous expression of the *A. fumigatus uge3* gene encoding a GalNAc-epimerase in *A. nidulans* results in the production of *A. fumigatus*-like GalNAc-rich GAG (32). Unlike wild-type *A. nidulans*, the GalNAc-rich GAG-producing strain of *A. nidulans* forms biofilm, produces extracellular matrix, and resists killing by neutrophils. The protective effects of GAG are dependent on NADPH oxidase and likely involve defense against NETs released through activation of this pathway. It has been hypothesized that GAG-mediated protection against NETs is mediated by electrostatic repulsion between this partially deacetylated cationic polysaccharide and cationic antimicrobial peptides or histones contained within NETs (32).

The immunomodulatory effects of GAG during biofilm formation are likely multifactorial. Hyphal-associated GAG masks β-glucans on the cell wall of hyphae (47, 128) and alters recognition by murine bone marrow-derived dendritic cells *in vitro* (47). Genetic disruption of GAG synthesis leads to increased pro-inflammatory cytokine release through Dectin-1 signaling (47). In a non-neutropenic murine model of pulmonary aspergillosis, genetic disruption of GAG synthesis results in production of a non-protective, hyper-inflammatory response marked by increased neutrophil recruitment (47). This observation suggests that GAG impairs neutrophil recruitment during biofilm growth and is consistent with the reports that soluble GAG can modulate immunity through induction of apoptosis in neutrophils and stimulation of anti-inflammatory IL-1Ra production by macrophages *in vitro* (49, 129). Further, *in vivo* studies are needed to evaluate the relative role of these functions of GAG in invasive and chronic *Aspergillus* infections.

Recent studies have begun to shed light on the mechanisms involved in NET release in response to fungi (32, 46, 93, 97, 124–126, 130). Specific ligands triggering this response to *Aspergillus* remain largely unknown, and how the extracellular matrix may influence these pathways is of great interest. For example, NET production is reduced in response to resting conidia when compared to hyphae (46). This inhibition is linked to RodA, a hydrophobin on the surface of conidia that masks pathogen-associated molecular patterns, including β-glucan (46, 48). As transcriptional analysis shows abundance of *RodA* during *A. fumigatus* biofilm growth when compared to planktonic conditions, it is interesting to postulate a role for RodA production in immune evasion during biofilm growth (131). Also, as melanin production has been described for some *Aspergillus* biofilms, investigation of a role for this immune modulator is also intriguing (4, 18, 33, 118).

## *Cryptococcus* Biofilms

### Biofilm Formation

*Cryptococcus* spp. are opportunistic environmental fungal pathogens that cause life-threatening meningoencephalitis, particularly in patients with suppressed immunity in the setting of HIV or organ transplantation (132). Following inhalation of spores from the environment, *C. neoformans* disseminates from the lungs, with a propensity for the central nervous system*. C. neoformans* also exhibits a predilection for artificial surfaces and forms biofilms on medical devices, such as cerebrospinal fluid shunts, vascular catheters, and prosthetic dialysis fistulae (133–136). These adherent communities are composed of yeast encased in an extracellular matrix (5, 41, 137). *In vitro, C. neoformans* biofilms mature in 24–48 h and display a multiple-drug-resistance phenotype (5).

### Matrix Production

*Cryptococcus neoformans* produces a protective polysaccharide capsule composed of glucuronoxylomannan (GXM), galactoxylomannan, and mannoprotein (137, 138). During biofilm growth, these capsular polysaccharides are shed into the surrounding milieu, ultimately providing extracellular matrix material for surface adhesion and cell–cell cohesion (5). Acapsular *C. neoformans* mutants are unable to form biofilms (5). Martinez and Casadevall identified GXM as the principle polysaccharide of the *C. neoformans* biofilm matrix (41). This polysaccharide has received the most attention due to its immunomodulatory properties and high abundance in the biofilm matrix (5, 138–141). However, biochemical analysis also shows the presence of sugars not found in GXM, suggesting that the biofilm matrix contains additional polysaccharides (41). Little is known about the structure of these polysaccharides and how they may influence immunity to *Cryptococcus* biofilms.

### Innate Immunity to *Cryptococcus* Biofilms

While studies have begun to dissect the impact of biofilm formation on immunity to *Cryptococcus*, much of this host–fungal interaction remains a mystery. Production of a GXM-rich extracellular matrix appears to be the key defense against host immunity. Genetic or antibody-mediated disruption of GXM impairs biofilm formation and diminishes virulence (5, 141). As a capsule polysaccharide, GXM is responsible for a multifaceted inhibition of neutrophil function, impeding chemotaxis, phagocytosis, NET production, and antifungal activity (138, 139, 142, 143). Similar mechanisms of diminished neutrophil function are anticipated in response to *C. neoformans* biofilms and may even be augmented given the high GXM content of biofilm matrix (41). Furthermore, capsular GXM can impair phagocytosis by monocytes and macrophages (138). However, it is unknown if phagocytosis would be an effective response against *Cryptococcus* biofilms, given the large structure of cohesive, aggregated yeast (41).

In addition to the immunomodulatory activity of the extracellular matrix, *C. neoformans* biofilms also resist antimicrobial host defenses. Compared to planktonic *C. neoformans*, biofilms tolerate higher concentrations of defensins, including PG-1, β-defensin-1, and β-defensin-3 (27). This resistance is even further augmented when biofilms are induced to produce melanin through l-Dopa supplementation. Biofilm formation also protects *C. neoformans* from oxidative stress induced by a variety of stimuli (27). Taken together, these studies show that *Cryptococcus* biofilms withstand innate immunity through both immune modulation and resistance to immune attack.

## Conclusion

Adoption of a biofilm lifestyle during fungal infection is increasingly recognized as a mechanism to avoid host immune attack and provide a protective niche. In this environment, the extracellular matrix can shield the fungal cell wall from host cellular recognition, modulating the immune response. In addition, the extracellular matrix can provide protection from antimicrobial defenses, such as defensins, oxidative stress, and NETs. Furthermore, biofilm formation produces an aggregated community that may resist engulfment by phagocytosis.

While it is clear that biofilm formation significantly influences immunity, studies are just beginning to shed light on the many mechanisms underlying this modulation of host response. As biofilms are heterogeneous structures with variations in architecture and composition based on their environmental niche, mechanisms impairing immunity likely vary among clinical biofilms. Therefore, inclusion of conditions closely mimicking the host and animal models of biofilm infection remains critical for future studies. While recent studies have revealed the influence of biofilm formation on the innate immune response, still little is known about how these structures may modulate adaptive immunity.

Fungal biofilms are among the most difficult infections to treat due to their high tolerance of antifungals and immune evasion strategies. The incidence of fungal biofilm infections is likely to rise given the growing number of patients with artificial medical devices and immunocompromising conditions. Anti-biofilm therapies are urgently needed. Understanding the dynamics of biofilm formation, matrix production, and how these processes induce resistance to multiple facets of the innate immune system may lead to biofilm-specific antifungal strategies.

## Author Contributions

JK, DS, and JN wrote the manuscript. JK and BS constructed the figures.

## Conflict of Interest Statement

The authors declare that the research was conducted in the absence of any commercial or financial relationships that could be construed as a potential conflict of interest.
